# Supplementary material for: Resolving the full spectrum of human genome variation using Linked-Reads
Source: Genome Res. 2019 Apr;29(4):635–45. doi: 10.1101/gr.234443.118 (PMC6442396; doi:10.1101/gr.234443.118)
Supplement: Supplemental Material [file supp_gr.234443.118_Supplemental_longranger-2.2.zip › longranger-2.2.tar.bz2!/lr2.2-public/lib/stan/cmdstan/stan/doc/api/html/autodiff_8hpp_source.html]

Stan: src/stan/agrad/autodiff.hpp Source File


|  |  |
| --- | --- |
|  | Stan  2.5.0  probability, sampling & optimization |


- Main Page
- Related Pages
- Namespaces
- Classes
- Files

- File List
- File Members

All Classes Namespaces Files Functions Variables Typedefs Enumerations Enumerator Friends Macros Pages

- src
- stan
- agrad

autodiff.hpp

Go to the documentation of this file.

1 #ifndef STAN\_\_AGRAD\_\_AUTO\_DIFF\_HPP

2 #define STAN\_\_AGRAD\_\_AUTO\_DIFF\_HPP

3

4 #include <stan/math/matrix/Eigen.hpp>

5 #include <stan/agrad/rev.hpp>

6 #include <stan/agrad/fwd.hpp>

7

8 namespace stan {

9

10  namespace agrad {

11

23  template <typename T, typename F>

24  void

25  derivative(const F& f,

26  const T& x,

27  T& fx,

28  T& dfx\_dx) {

29  fvar<T> x\_fvar = fvar<T>(x,1.0);

30  fvar<T> fx\_fvar = f(x\_fvar);

31  fx = fx\_fvar.val\_;

32  dfx\_dx = fx\_fvar.d\_;

33  }

34

47  template <typename T, typename F>

48  void

49  partial\_derivative(const F& f,

50  const Eigen::Matrix<T,Eigen::Dynamic,1>& x,

51  int n,

52  T& fx,

53  T& dfx\_dxn) {

54  Eigen::Matrix<fvar<T>,Eigen::Dynamic,1> x\_fvar(x.size());

55  for (int i = 0; i < x.size(); ++i)

56  x\_fvar(i) = fvar<T>(x(i),i==n);

57  fvar<T> fx\_fvar = f(x\_fvar);

58  fx = fx\_fvar.val\_;

59  dfx\_dxn = fx\_fvar.d\_;

60  }

61

91  template <typename F>

92  void

93  gradient(const F& f,

94  const Eigen::Matrix<double,Eigen::Dynamic,1>& x,

95  double& fx,

96  Eigen::Matrix<double,Eigen::Dynamic,1>& grad\_fx) {

97  using stan::agrad::var;

98  start\_nested();

99  try {

100  Eigen::Matrix<var,Eigen::Dynamic,1> x\_var(x.size());

101  for (int i = 0; i < x.size(); ++i)

102  x\_var(i) = x(i);

103  var fx\_var = f(x\_var);

104  fx = fx\_var.val();

105  grad\_fx.resize(x.size());

106  stan::agrad::grad(fx\_var.vi\_);

107  for (int i = 0; i < x.size(); ++i)

108  grad\_fx(i) = x\_var(i).adj();

109  } catch (const std::exception& /\*e\*/) {

110  stan::agrad::recover\_memory\_nested();

111  throw;

112  }

113  stan::agrad::recover\_memory\_nested();

114  }

115  template <typename T, typename F>

116  void

117  gradient(const F& f,

118  const Eigen::Matrix<T,Eigen::Dynamic,1>& x,

119  T& fx,

120  Eigen::Matrix<T,Eigen::Dynamic,1>& grad\_fx) {

121  Eigen::Matrix<fvar<T>,Eigen::Dynamic,1> x\_fvar(x.size());

122  grad\_fx.resize(x.size());

123  for (int i = 0; i < x.size(); ++i) {

124  for (int k = 0; k < x.size(); ++k)

125  x\_fvar(k) = fvar<T>(x(k),k==i);

126  fvar<T> fx\_fvar = f(x\_fvar);

127  if (i == 0) fx = fx\_fvar.val\_;

128  grad\_fx(i) = fx\_fvar.d\_;

129  }

130  }

131

132

133  template <typename F>

134  void

135  jacobian(const F& f,

136  const Eigen::Matrix<double,Eigen::Dynamic,1>& x,

137  Eigen::Matrix<double,Eigen::Dynamic,1>& fx,

138  Eigen::Matrix<double,Eigen::Dynamic,Eigen::Dynamic>& J) {

139  using Eigen::Matrix; using Eigen::Dynamic;

140  using stan::agrad::var;

141  start\_nested();

142  try {

143  Matrix<var,Dynamic,1> x\_var(x.size());

144  for (int k = 0; k < x.size(); ++k)

145  x\_var(k) = x(k);

146  Matrix<var,Dynamic,1> fx\_var = f(x\_var);

147  fx.resize(fx\_var.size());

148  for (int i = 0; i < fx\_var.size(); ++i)

149  fx(i) = fx\_var(i).val();

150  J.resize(x.size(), fx\_var.size());

151  for (int i = 0; i < fx\_var.size(); ++i) {

152  if (i > 0)

153  set\_zero\_all\_adjoints();

154  grad(fx\_var(i).vi\_);

155  for (int k = 0; k < x.size(); ++k)

156  J(k,i) = x\_var(k).adj();

157  }

158  } catch (const std::exception& e) {

159  stan::agrad::recover\_memory\_nested();

160  throw;

161  }

162  stan::agrad::recover\_memory\_nested();

163  }

164  template <typename T, typename F>

165  void

166  jacobian(const F& f,

167  const Eigen::Matrix<T,Eigen::Dynamic,1>& x,

168  Eigen::Matrix<T,Eigen::Dynamic,1>& fx,

169  Eigen::Matrix<T,Eigen::Dynamic,Eigen::Dynamic>& J) {

170  using Eigen::Matrix; using Eigen::Dynamic;

171  using stan::agrad::fvar;

172  Matrix<fvar<T>,Dynamic,1> x\_fvar(x.size());

173  for (int i = 0; i < x.size(); ++i) {

174  for (int k = 0; k < x.size(); ++k)

175  x\_fvar(k) = fvar<T>(x(k), i == k);

176  Matrix<fvar<T>,Dynamic,1> fx\_fvar

177  = f(x\_fvar);

178  if (i == 0) {

179  J.resize(x.size(),fx\_fvar.size());

180  fx.resize(fx\_fvar.size());

181  for (int k = 0; k < fx\_fvar.size(); ++k)

182  fx(k) = fx\_fvar(k).val\_;

183  }

184  for (int k = 0; k < fx\_fvar.size(); ++k) {

185  J(i,k) = fx\_fvar(k).d\_;

186  }

187  }

188  }

189

190

191  // time O(N^2); space O(N^2)

192  template <typename F>

193  void

194  hessian(const F& f,

195  const Eigen::Matrix<double,Eigen::Dynamic,1>& x,

196  double& fx,

197  Eigen::Matrix<double,Eigen::Dynamic,1>& grad,

198  Eigen::Matrix<double,Eigen::Dynamic,Eigen::Dynamic>& H) {

199  start\_nested();

200  try {

201  H.resize(x.size(), x.size());

202  grad.resize(x.size());

203  for (int i = 0; i < x.size(); ++i) {

204  Eigen::Matrix<fvar<var>, Eigen::Dynamic, 1> x\_fvar(x.size());

205  for (int j = 0; j < x.size(); ++j)

206  x\_fvar(j) = fvar<var>(x(j),i==j);

207  fvar<var> fx\_fvar = f(x\_fvar);

208  grad(i) = fx\_fvar.d\_.val();

209  if (i == 0) fx = fx\_fvar.val\_.val();

210  stan::agrad::grad(fx\_fvar.d\_.vi\_);

211  for (int j = 0; j < x.size(); ++j)

212  H(i,j) = x\_fvar(j).val\_.adj();

213  }

214  } catch (const std::exception& e) {

215  stan::agrad::recover\_memory\_nested();

216  throw;

217  }

218  stan::agrad::recover\_memory\_nested();

219  }

220  // time O(N^3); space O(N^2)

221  template <typename T, typename F>

222  void

223  hessian(const F& f,

224  const Eigen::Matrix<T,Eigen::Dynamic,1>& x,

225  T& fx,

226  Eigen::Matrix<T,Eigen::Dynamic,1>& grad,

227  Eigen::Matrix<T,Eigen::Dynamic,Eigen::Dynamic>& H) {

228  H.resize(x.size(), x.size());

229  grad.resize(x.size());

230  Eigen::Matrix<fvar<fvar<T> >,Eigen::Dynamic,1> x\_fvar(x.size());

231  for (int i = 0; i < x.size(); ++i) {

232  for (int j = i; j < x.size(); ++j) {

233  for (int k = 0; k < x.size(); ++k)

234  x\_fvar(k) = fvar<fvar<T> >(fvar<T>(x(k),j==k),

235  fvar<T>(i==k,0));

236  fvar<fvar<T> > fx\_fvar = f(x\_fvar);

237  if (j == 0)

238  fx = fx\_fvar.val\_.val\_;

239  if (i == j)

240  grad(i) = fx\_fvar.d\_.val\_;

241  H(i,j) = fx\_fvar.d\_.d\_;

242  H(j,i) = H(i,j);

243  }

244  }

245  }

246

247

248  // aka directional derivative (not length normalized)

249  // T2 must be assignable to T1

250  template <typename T1, typename T2, typename F>

251  void

252  gradient\_dot\_vector(const F& f,

253  const Eigen::Matrix<T1,Eigen::Dynamic,1>& x,

254  const Eigen::Matrix<T2,Eigen::Dynamic,1>& v,

255  T1& fx,

256  T1& grad\_fx\_dot\_v) {

257  using stan::agrad::fvar;

258  using stan::agrad::var;

259  using Eigen::Matrix;

260  using Eigen::Dynamic;

261  Matrix<fvar<T1>,Dynamic,1> x\_fvar(x.size());

262  for (int i = 0; i < x.size(); ++i)

263  x\_fvar(i) = fvar<T1>(x(i),v(i));

264  fvar<T1> fx\_fvar = f(x\_fvar);

265  fx = fx\_fvar.val\_;

266  grad\_fx\_dot\_v = fx\_fvar.d\_;

267  }

268

269

270

271

272  template <typename F>

273  void

274  hessian\_times\_vector(const F& f,

275  const Eigen::Matrix<double,Eigen::Dynamic,1>& x,

276  const Eigen::Matrix<double,Eigen::Dynamic,1>& v,

277  double& fx,

278  Eigen::Matrix<double,Eigen::Dynamic,1>& Hv) {

279  using stan::agrad::fvar;

280  using stan::agrad::var;

281  using Eigen::Matrix;

282  using Eigen::Dynamic;

283  start\_nested();

284  try {

285  Matrix<var,Dynamic,1> x\_var(x.size());

286  for (int i = 0; i < x\_var.size(); ++i)

287  x\_var(i) = x(i);

288  var fx\_var;

289  var grad\_fx\_var\_dot\_v;

290  gradient\_dot\_vector(f,x\_var,v,fx\_var,grad\_fx\_var\_dot\_v);

291  fx = fx\_var.val();

292  stan::agrad::grad(grad\_fx\_var\_dot\_v.vi\_);

293  Hv.resize(x.size());

294  for (int i = 0; i < x.size(); ++i)

295  Hv(i) = x\_var(i).adj();

296  } catch (const std::exception& e) {

297  stan::agrad::recover\_memory\_nested();

298  throw;

299  }

300  stan::agrad::recover\_memory\_nested();

301  }

302  template <typename T, typename F>

303  void

304  hessian\_times\_vector(const F& f,

305  const Eigen::Matrix<T,Eigen::Dynamic,1>& x,

306  const Eigen::Matrix<T,Eigen::Dynamic,1>& v,

307  T& fx,

308  Eigen::Matrix<T,Eigen::Dynamic,1>& Hv) {

309  using Eigen::Matrix;

310  using Eigen::Dynamic;

311  Matrix<T,Dynamic,1> grad;

312  Matrix<T,Dynamic,Dynamic> H;

313  hessian(f,x,fx,grad,H);

314  Hv = H \* v;

315  }

316

317  // FIXME: add other results that are easy to extract

318  // // N \* (fwd(2) + bk)

319  template <typename F>

320  void

321  grad\_tr\_mat\_times\_hessian(

322  const F& f,

323  const Eigen::Matrix<double,Eigen::Dynamic,1>& x,

324  const Eigen::Matrix<double,Eigen::Dynamic,Eigen::Dynamic>& M,

325  Eigen::Matrix<double,Eigen::Dynamic,1>& grad\_tr\_MH) {

326  using Eigen::Matrix;

327  using Eigen::Dynamic;

328  start\_nested();

329  try {

330

331  grad\_tr\_MH.resize(x.size());

332

333  Matrix<var,Dynamic,1> x\_var(x.size());

334  for (int i = 0; i < x.size(); ++i)

335  x\_var(i) = x(i);

336

337  Matrix<fvar<var>,Dynamic,1> x\_fvar(x.size());

338

339  var sum(0.0);

340  Matrix<double,Dynamic,1> M\_n(x.size());

341  for (int n = 0; n < x.size(); ++n) {

342  for (int k = 0; k < x.size(); ++k)

343  M\_n(k) = M(n,k);

344  for (int k = 0; k < x.size(); ++k)

345  x\_fvar(k) = fvar<var>(x\_var(k), k == n);

346  fvar<var> fx;

347  fvar<var> grad\_fx\_dot\_v;

348  gradient\_dot\_vector<fvar<var>,double>(f,x\_fvar,M\_n,fx,grad\_fx\_dot\_v);

349  sum += grad\_fx\_dot\_v.d\_;

350  }

351

352  stan::agrad::grad(sum.vi\_);

353  for (int i = 0; i < x.size(); ++i)

354  grad\_tr\_MH(i) = x\_var(i).adj();

355  } catch (const std::exception& e) {

356  stan::agrad::recover\_memory\_nested();

357  throw;

358  }

359  stan::agrad::recover\_memory\_nested();

360  }

361

362  }

363 }

364 #endif

stan::agrad::gradient

void gradient(const F &f, const Eigen::Matrix< double, Eigen::Dynamic, 1 > &x, double &fx, Eigen::Matrix< double, Eigen::Dynamic, 1 > &grad\_fx)

Calculate the value and the gradient of the specified function at the specified argument.

**Definition:** autodiff.hpp:93

stan::agrad::set\_zero\_all\_adjoints

static void set\_zero\_all\_adjoints()

Reset all adjoint values in the stack to zero.

**Definition:** chainable.hpp:87

stan::agrad::start\_nested

static void start\_nested()

Record the current position so that recover\_memory\_nested() can find it.

**Definition:** var\_stack.hpp:96

stan::agrad::var::val

double val() const

Return the value of this variable.

**Definition:** var.hpp:209

stan::agrad::fvar

**Definition:** fvar.hpp:13

fwd.hpp

stan::agrad::grad

static void grad(chainable \*vi)

Compute the gradient for all variables starting from the specified root variable implementation.

**Definition:** chainable.hpp:110

stan::agrad::partial\_derivative

void partial\_derivative(const F &f, const Eigen::Matrix< T, Eigen::Dynamic, 1 > &x, int n, T &fx, T &dfx\_dxn)

Return the partial derivative of the specified multiivariate function at the specified argument...

**Definition:** autodiff.hpp:49

stan::agrad::hessian\_times\_vector

void hessian\_times\_vector(const F &f, const Eigen::Matrix< double, Eigen::Dynamic, 1 > &x, const Eigen::Matrix< double, Eigen::Dynamic, 1 > &v, double &fx, Eigen::Matrix< double, Eigen::Dynamic, 1 > &Hv)

**Definition:** autodiff.hpp:274

stan::agrad::sum

fvar< T > sum(const Eigen::Matrix< fvar< T >, R, C > &m)

**Definition:** sum.hpp:14

stan::agrad::var::vi\_

vari \* vi\_

Pointer to the implementation of this variable.

**Definition:** var.hpp:40

stan::agrad::derivative

void derivative(const F &f, const T &x, T &fx, T &dfx\_dx)

Return the derivative of the specified univariate function at the specified argument.

**Definition:** autodiff.hpp:25

rev.hpp

stan::agrad::grad\_tr\_mat\_times\_hessian

void grad\_tr\_mat\_times\_hessian(const F &f, const Eigen::Matrix< double, Eigen::Dynamic, 1 > &x, const Eigen::Matrix< double, Eigen::Dynamic, Eigen::Dynamic > &M, Eigen::Matrix< double, Eigen::Dynamic, 1 > &grad\_tr\_MH)

**Definition:** autodiff.hpp:321

Eigen.hpp

stan::agrad::var

Independent (input) and dependent (output) variables for gradients.

**Definition:** var.hpp:27

stan::math::e

double e()

Return the base of the natural logarithm.

**Definition:** constants.hpp:86

stan::agrad::jacobian

void jacobian(const F &f, const Eigen::Matrix< double, Eigen::Dynamic, 1 > &x, Eigen::Matrix< double, Eigen::Dynamic, 1 > &fx, Eigen::Matrix< double, Eigen::Dynamic, Eigen::Dynamic > &J)

**Definition:** autodiff.hpp:135

stan::agrad::gradient\_dot\_vector

void gradient\_dot\_vector(const F &f, const Eigen::Matrix< T1, Eigen::Dynamic, 1 > &x, const Eigen::Matrix< T2, Eigen::Dynamic, 1 > &v, T1 &fx, T1 &grad\_fx\_dot\_v)

**Definition:** autodiff.hpp:252

stan::agrad::fvar::d\_

T d\_

**Definition:** fvar.hpp:16

stan::agrad::recover\_memory\_nested

static void recover\_memory\_nested()

Recover only the memory used for the top nested call.

**Definition:** var\_stack.hpp:72

stan::agrad::hessian

void hessian(const F &f, const Eigen::Matrix< double, Eigen::Dynamic, 1 > &x, double &fx, Eigen::Matrix< double, Eigen::Dynamic, 1 > &grad, Eigen::Matrix< double, Eigen::Dynamic, Eigen::Dynamic > &H)

**Definition:** autodiff.hpp:194

stan::agrad::fvar::val\_

T val\_

**Definition:** fvar.hpp:15


---

[ Stan Home Page ]

*© 2011–2014,
Stan Development Team.*
